# Supplementary material for: Estimating total human deaths from rabies: A statistical framework leveraging reports of rabies transmission from deceased organ and tissue donors
Source: PLoS Negl Trop Dis. 2026 Sep 21;20(9):e0014731. doi: 10.1371/journal.pntd.0014731 (PMC13592719; doi:10.1371/journal.pntd.0014731)

Mongolia (canine−rabies−endemic)

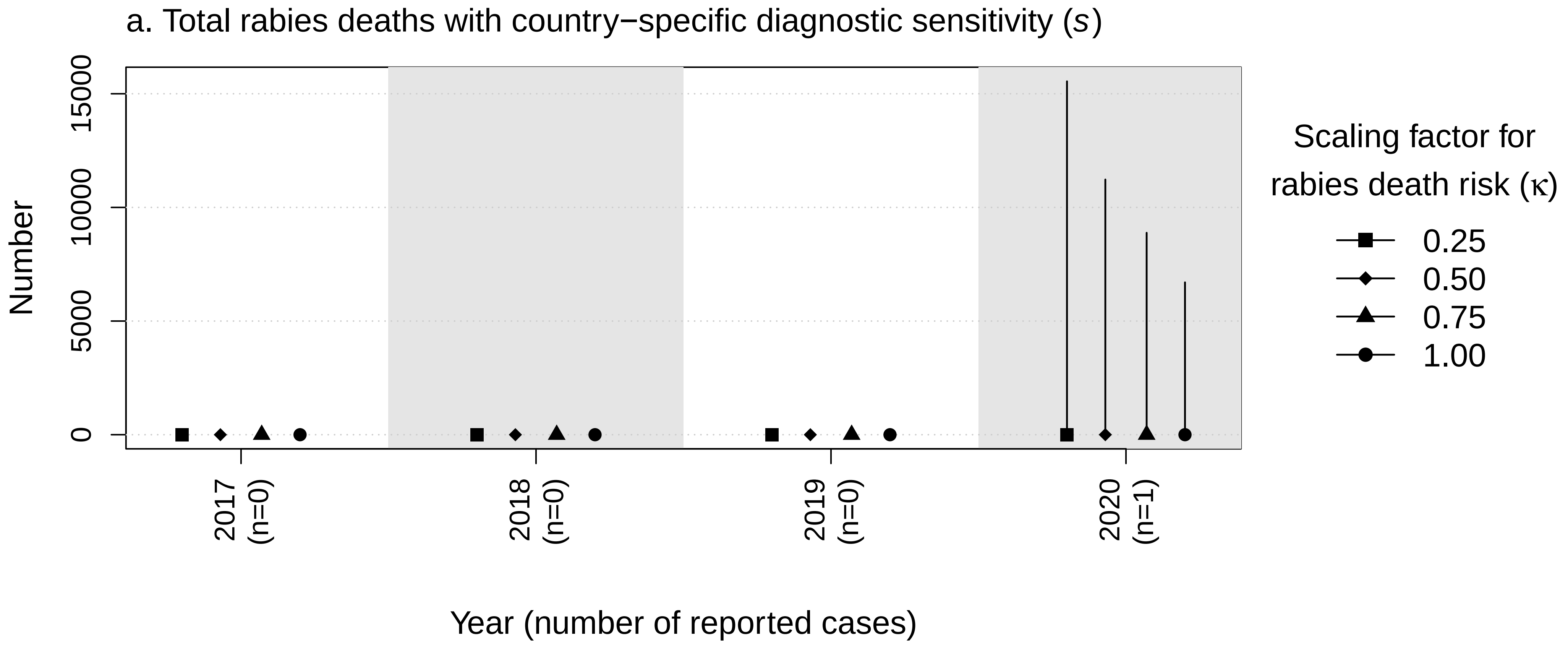

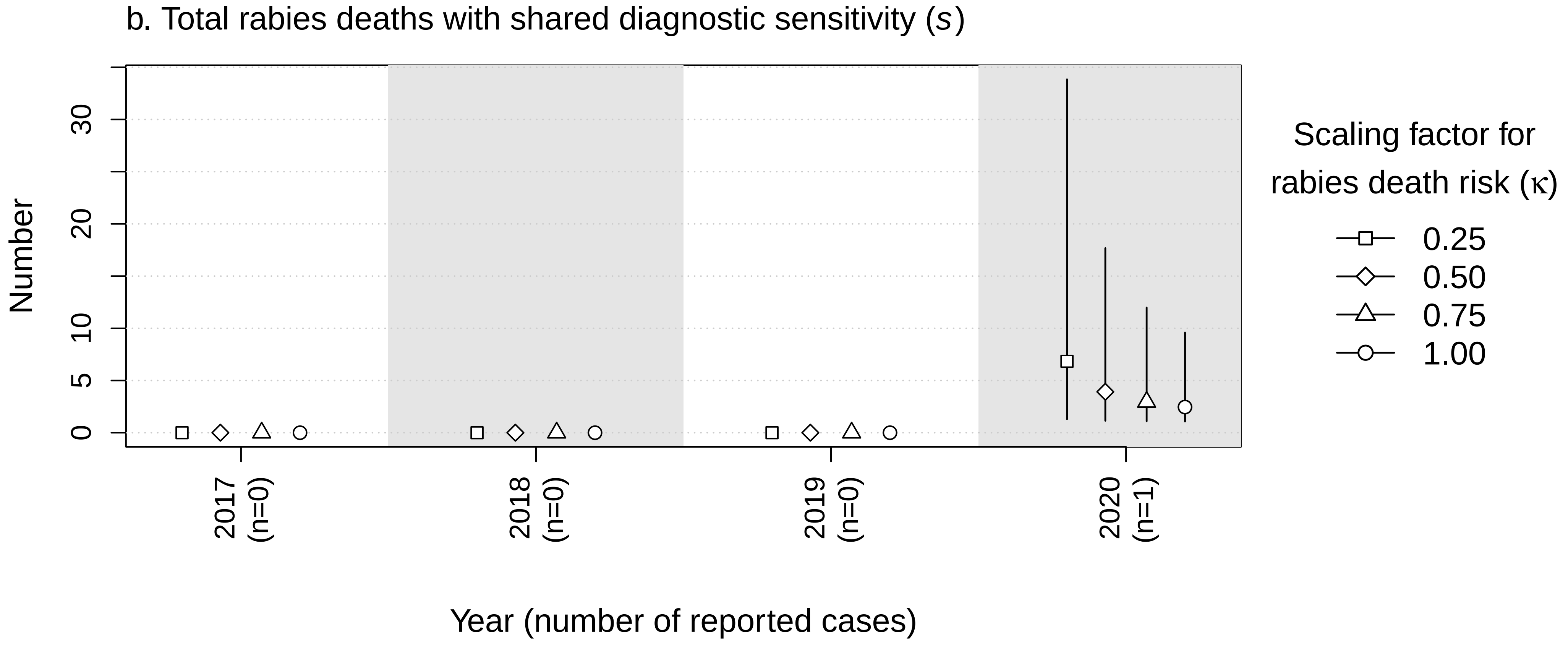

Supplement: Supplementary file 6 [file pntd.0014731.s005.zip › Result_A/Mongolia/Figure_S3_Mongolia.pdf]
